# An Eye on Animacy and Intention

**DOI:** 10.3389/fpsyg.2016.00829

**Published:** 2016-06-03

**Authors:** Dorothea U. Martin, Conrad Perry, Jordy H. Kaufman

**Affiliations:** Swinburne Babylab, Brain & Psychological Sciences Research Centre, Swinburne University of TechnologyHawthorn, VIC, Australia

**Keywords:** eye-gaze, intention, animacy, infancy, social cognition

Hamlin (2015) argued that the human capacity for social evaluation begins to emerge just a few months after birth. This claim derives from studies using unfulfilled goal scenarios, with infants as young as 3 months of age (see Hamlin, 2013). In a typical “manual choice” experiment, infants are habituated to multiple helping and hindering events. For example, a protagonist may first fail to achieve a goal such as climbing a hill. Then, another character pushes the protagonist either up the hill (the “Helper”) or back down the hill (the “Hinderer”). Following habituation, infants are presented with the Helper and Hinderer. By 4.5 months of age, infants more often choose to reach for the Helper than the Hinderer. These results are interpreted as evidence for an early developing sense of morality (Hamlin, 2013, 2014; c.f. Cowell and Decety, 2015; Salvadori et al., 2015; Scola et al., 2015).

One potential issue with Hamlin et al.'s (2007) study, proposed by Scarf et al. (2012), is that the Helper events were confounded with an act of bouncing when the protagonist reached the hilltop. The current paper, however, showed that this was not the case, and that the direction of the protagonist's eye-gaze was the important factor. In this case, children chose the Helper over the Hinderer more often when the protagonist gazed at the hilltop. Hamlin argued that eye-gaze shows intent and that infants' understanding of intent allows them to correctly represent the behavior of the character as helping or hindering.

Regardless of whether eye-gaze served as a cue to intention, it is not surprising that gaze could have profound effects on infants' preferences. Eye contact and following others' gaze is a crucial part of our daily interactions. This sensitivity to eyes appears to be highly adaptive (Baron-Cohen, 1995; Langten et al., 2000). Even at birth when infants have poor visual acuity and ocular control, they have a preference for open over closed eyes (Batki et al., 2000) and direct over averted gaze (Farroni et al., 2002, 2006). At 6 months of age, an important milestone occurs as infants begin to follow others' eye gaze to targets inside their current field of view (D'Entremont et al., 1997; Gredebäck et al., 2008). The sensitivity to eye-gaze is assumed to have its roots in the evolution of the morphology of the human eye—which has an unusually high contrast between iris, sclera, and surrounding skin, enabling the detection of gaze direction from a great distance (Kobayashi and Kohshima, 1997, 2001; Tomasello et al., 2007).

Although, the eye-gaze is clearly important in the task of Hamlin, it is not entirely clear why. One possibility is that eye-gaze helps the understanding of intentions. Such an explanation has been proposed by Baron-Cohen (1994, 1995) who suggested that humans have an eye-direction detector that detects eye-like stimuli and computes gaze direction. This mechanism in conjunction with an Intentionality Detector and Shared Attention Mechanism trigger the development of the Theory-of-Mind-Mechanism (ToMM), which allows mental states, such as people's intentions, to be inferred. The actual age at which intentions can be understood, however, is debated with both Carpenter et al. (1998) and Tomasello et al. (1993, 2005) suggesting that such a skill does not emerge until the age of one, which is older than the children in Hamlin's (2015) study.

An alternative possibility is that infants prefer an event where someone “alive” is helped. In this case, infants may be responding to consistent animate action, regardless of any understanding of intentions (Tomasello et al., 2005). In particular, gaze may have been a cue for animacy in Hamlin's (2015) study when the climber's gaze was fixed on the hilltop (see Looser and Wheatley, 2010; Opfer and Gelman, 2010). Thus, infants may have preferred the Helper over the Hinderer in the “animate” condition, as only the Helper facilitated movements that were consistent with the previous event of ascending the first incline. If this is correct, then one would not need to assume infants of 6–11 months must understand the intentions of the puppet to do the task.

Whether infants' preference for Helpers is based on an innate morality remains an open question. Nevertheless, Hamlin's (2015) experiments provided novel insights showing the importance of eye gaze for infants' choices. Because one has to understand the other's intention in order to act prosocially, future research addressing the question of whether young infants are truly able to infer from the Protagonist's gaze to its goals or if infants' choices are solely based on their predictions of future animate action would be of interest.

## Author contributions

All authors listed, have made substantial, direct and intellectual contribution to the work, and approved it for publication.

### Conflict of interest statement

The authors declare that the research was conducted in the absence of any commercial or financial relationships that could be construed as a potential conflict of interest.
